# Gut Microbiota Profiles of Treated Metabolic Syndrome Patients and their Relationship with Metabolic Health

**DOI:** 10.1038/s41598-020-67078-3

**Published:** 2020-06-22

**Authors:** Montree Wutthi-in, Supapon Cheevadhanarak, Sakawdaurn Yasom, Sasiwan Kerdphoo, Parameth Thiennimitr, Arintaya Phrommintikul, Nipon Chattipakorn, Weerayuth Kittichotirat, Siriporn Chattipakorn

**Affiliations:** 1 Bioinformatics and Systems Biology Program, School of Bioresources and Technology and School of Information Technology, King Mongkut’s University of Technology Thonburi, Bangkok, 10150 Thailand; 2 Systems Biology and Bioinformatics Research Group, Pilot Plant Development and Training Institute, King Mongkut’s University of Technology Thonburi, Bangkok, 10150 Thailand; 3 School of Bioresources and Technology, King Mongkut’s University of Technology Thonburi, Bangkok, 10150 Thailand; 4 Cardiac Electrophysiology Research and Training Center, Faculty of Medicine, Chiang Mai University, Chiang Mai, 50200 Thailand; 5 Center of Excellence in Cardiac Electrophysiology Research, Faculty of Medicine, Chiang Mai University, Chiang Mai, 50200 Thailand; 6 Department of Microbiology, Faculty of Medicine, Chiang Mai University, Chiang Mai, 50200 Thailand; 7 Department of Internal Medicine, Faculty of Medicine, Chiang Mai University, Chiang Mai, 50200 Thailand; 8 Department of Oral Biology and Diagnostic Sciences, Faculty of Dentistry, Chiang Mai University, Chiang Mai, 50200 Thailand

**Keywords:** Metabolic syndrome, Preclinical research

## Abstract

Metabolic syndrome (MetS) has become a worldwide health issue. Recent studies reveal that the human gut microbiota exerts a significant role in the pathogenesis of this disease. While drug treatments may greatly improve metabolic symptoms, little is known about the gut microbiota composition of these treated MetS patients. This study aimed to characterize the gut microbiota composition of treated-MetS patients and analyse the possibility of using gut microbiota as an indicator of metabolic conditions. 16S rRNA metagenomic sequencing approach was used to profile gut microbiota of 111 treated MetS patients from The Cohort of patients at a high Risk of Cardiovascular Events (CORE)-Thailand registry. Our results show that the gut microbiota profiles of MetS patients are diverse across individuals, but can be classified based on their similarity into three groups or enterotypes. We also showed several associations between species abundance and metabolic parameters that are enterotype specific. These findings suggest that information on the gut microbiota can be useful for assessing treatment options for MetS patients. In addition, any correlations between species abundance and human properties are likely specific to each microbial community.

## Introduction

Metabolic syndrome (MetS) is characterized by a cluster of metabolic symptoms including obesity, hypertension, dyslipidemia, hyperglycemia, and insulin resistance^1^. People with MetS have an increased risk of mortality from cardiovascular and cerebrovascular diseases, as well as being at greater risk of neurodegeneration^2^. The criteria for diagnosing MetS are designated by values for obesity (e.g. waist circumference or body mass index (BMI)), triglyceride, high-density lipoprotein cholesterol (HDL-C), hypertension, and urine or blood albumin^3,4^. According to American Heart Association criteria, approximately 35% of adults in United States and 50% of people who are older than 60 years have metabolic syndrome^3,5^. For decades a great deal of research has been undertaken to better understand the factors that can lead to MetS. The development of MetS may be due to genetic factors as well as lifestyle including sedentary habits and high-fat/high-sugar dietary consumption^6^. Several recent studies have demonstrated that the human gut microbiota, the complex microbial community living inside the human gastrointestinal tract, plays a significant role in the pathogenesis of MetS^7–11^. Previous studies have shown that interactions between gut microbiota and host metabolism could result in either promotion or protection of the host from metabolic disorders^12–17^. In general, human and mice share two similar major phyla within their gut microbiota, specifically Firmicutes, and Bacteroidetes^18^. The development of obesity has been shown to be associated with an increased Firmicutes/Bacteroidetes (F/B) ratio and higher Proteobacteria levels in the gastrointestinal tract^19–22^. However, recent characterization studies based on large cohorts also demonstrated inconsistent findings where F/B ratios were not significantly different among subjects with different body weights^10,23^. Besides that, Arumugam *et al*. showed that human gut microbiota variation can be stratified into a limited number of common microbial abundance profiles, called enterotypes, that might respond differently to external factors such as diets or drug intakes^24^. While such stratification has been demonstrated to be useful in the analysis human gut microbiota, the result relies heavily on clustering criteria used in each study (e.g. choice of taxonomic levels, clustering algorithms, and distance functions) and therefore standardization of this approach is still an active area of research^25^.

Clinically, a patient is considered to have MetS when three or more of the following five conditions exist, which are (i) waist circumference ≥90 cm in men or ≥80 cm in women, (ii) blood pressure ≥130/85 mmHg, (iii) triglycerides ≥150 mg/dl, (iv) HDL-C < 40 mg/dl in men or <50 mg/dl in women, and (v) fasting glucose ≥100 mg/dl^26^. Various drugs are usually administered to treat MetS patients based on their metabolic symptoms. These include, for instance, Angiotensin converting enzyme inhibitor, Beta blocker, Calcium channel blocker, and Angiotensin receptor blocker for the treatment of high blood pressure, Statin, Fibrate, Niacin, and Ezetimibe for lowering lipid, Insulin, Sulfonyl urea, Metformin, Thiazolidinedione, and DPP4 inhibitor for lowering glucose, and Aspirin, Clopidogrel, and Warfarin as antithrombotic agents. While these drugs are very effective in improving patient outcomes in term of metabolic parameters, little is known about the condition of the gut microbiota of these treated patients. To this end, we hypothesize that common patterns of gut microbiota abundance or enterotypes exist among treated MetS patients and knowledge of these enterotypes may be useful for physicians to detect gut dysbiosis as well as understand the associations between microbial abundance and metabolic parameters. Therefore, the present study aimed to characterize the gut microbiota of clinically-treated MetS patients and identify existing enterotypes as well as relationship between microbiota profiles and metabolic outcomes of these patients.

## Results

### The demographic data of enrolled metabolic syndrome patients and metagenomic sequencing results

**Figure 1:**
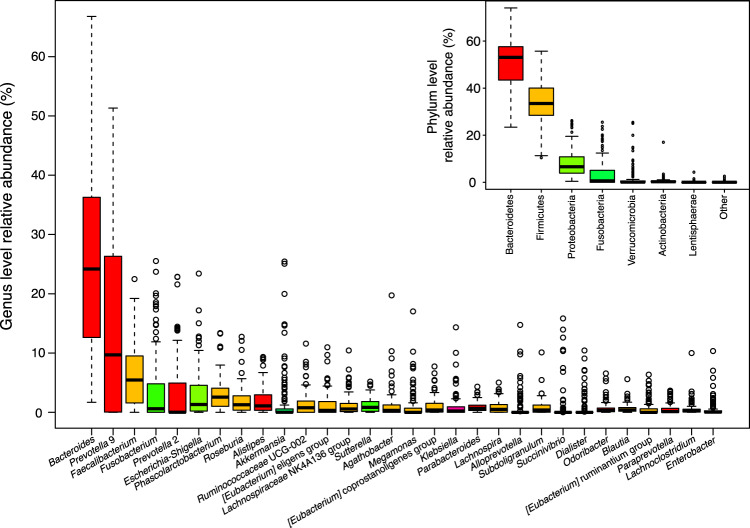
Box plots showing distributions of genera and phyla relative abundances identified across 111 metabolic syndrome patients. Only the top 30 genera in terms of relative abundance are shown in the plot. Relationship between each phylum and respective genera are shown using the same color bar.

A total of 111 clinically-treated MetS patients were recruited into this study. Table 1 shows the demographic data of MetS patients together with some their clinical parameters. A summary of gut microbiota metagenomic sequencing result of these patients can be found in Supplementary Fig. S1 and Table S1. Briefly, the sequencing of the V3–V4 hypervariable region of 16S rRNA gene produced between 36,400 and 83,728 paired-end reads that are 250 bases in length for each sample (mean number of reads of 66,557 ± 8,460). After preprocessing and amplicon sequence variant (ASV) identification, the number of remaining sequences for each sample was between 17,057 and 44,761 reads (mean number of reads of 30,797 ± 5,239) with a total of 8,754 different ASVs assigned across all samples. Some of the most abundant bacterial phyla found within these samples were Bacteroidetes, Firmicutes, Proteobacteria, Fusobacteria, and Verrucomicrobia as summarized in the inset of Fig. 1 where the relative abundance values of each taxa for all samples provided in Supplementary Data 1. Also shown in Fig. 1, these common phyla appeared to be made up of genera including *Bacteriodes*, *Prevotella 9*, *Faecalibaterium, Fusobacterium, Prevotella 2*, and *Escherichia-Shigella* as displayed by the same color bar. While *Bacteroides* was the found in high relative abundance in most samples, its presence also exhibited the greatest variation in terms of relative abundance, which is consistent with data reported in previous studies^24,27^.

**Table 1:** Sample demographic data of metabolic syndrome patients (N = 111) in this study. Categorical variables are expressed as frequency and percentage. Continuous variables are expressed as mean ± standard deviation.

Parameters	Values
Male (%)	49 (44.14)
Age (years)	64.297 ± 8.419
Height (cm)	157.486 ± 7.352
Weight (kg)	68.358 ± 15.252
BMI (kg/m^2^)	27.514 ± 5.764
Waist circumference (cm^2^)	94.935 ± 13.394
Obese (%)	32 (28.83)
Hypertension (%)	97 (87.4)
Diabetes mellitus (%)	88 (79.28)
Chronic Kidney Disease (%)	23 (20.72)
Dyslipidemia (%)	96 (84.49)
Coronary artery disease (%)	35 (31.5)
Cerebrovascular disease (%)	29 (26.1)
Smoke frequency (days per week)	0.405 ± 0.578
Alcohol consumption (days per week)	0.486 ± 0.785
High-density lipoprotein cholesterol (mg/dL)	49.667 ± 16.771
Low-Density lipoprotein cholesterol (mg/dL)	94.467 ± 36.188
Very-low-density lipoprotein cholesterol (mg/dL)	28.111 ± 15.877
Triglyceride (mg/dL)	133.408 ± 74.089
Glucose (mg/dL)	130.140 ± 44.771
Hemoglobin A1C (mg/dL)	7.159 ± 1.463
Fibroblast growth factor 21 (pg/mL)	389.794 ± 508.598
Insulin (mg/dL)	7.988 ± 8.931
Homeostatic Model Assessment score	2.600 ± 3.097

### Variation of gut microbiota composition among metabolic syndrome patients

**Figure 2:**
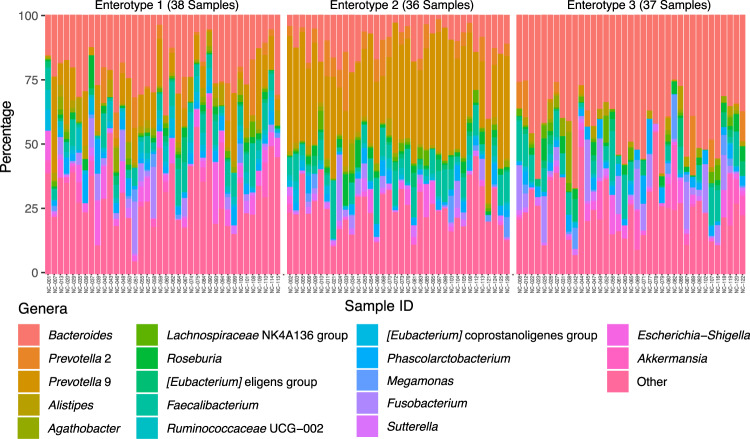
Genus relative abundance profiles of 111 metabolic syndrome patients separated by their enterotype assignments. Genera that exhibit less than 1 percent abundance across the samples are combined and shown as other.

**Figure 3:**
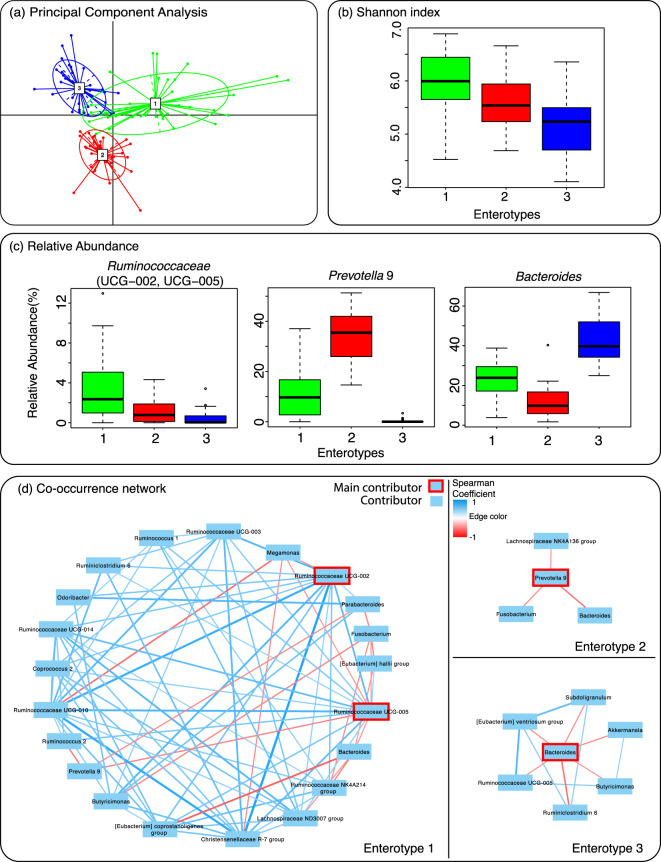
Variations in gut microbiota composition of metabolic syndrome patients. (**a**) Grouping of microbiota profiles based on similarity in species compositions resulted in 3 clusters or enterotypes as visualized by principal component analysis. (**b**) Diversity analysis using index for each enterotype of metabolic syndrome patients. (**c**) Relative abundance of main contributor in each enterotype. (**d**) Co-occurrence network of the main contributor(s) and other genera in each enterotype.

To better understand the gut microbiota variation among treated MetS patients, we first grouped their gut microbiota profiles based on the genus relative abundance similarity using a multidimensional clustering algorithm. Our clustering result revealed that gut microbiota profile of 111 MetS patients can be optimally classified into three groups or enterotypes that share similar genus abundance profiles (Supplementary Fig. S2). Specifically, enterotype 1, 2, and 3 could be assigned to 38, 36, and 37 patients respectively as shown by Fig. 2 and Supplementary Table S2. Principal component analysis showed that while most samples from each enterotype were well separated, a small number of samples appeared to be overlapping between enterotypes indicating that some microbiota profiles may possibly be classified to more than one enterotypes (Fig. 3a). The alpha diversity analysis using Shannon index showed that enterotype 1 exhibited the highest diversity when compared to other enterotypes (p < 0.001) as shown in Fig. 3b. Differential abundance analysis using ANalysis of COmposition of Microbiomes (ANCOM)^28^ showed that *Ruminococaceae* (UCG-002, UCG-005), *Prevotella* 9, and *Bacteroides* were significantly different between enterotypes (Supplementary Data 2) and their abundance variations can be used to identify enterotype 1, 2, and 3 respectively as shown by Fig. 3c. Additionally, differential abundance analysis using Linear discriminant analysis Effect Size (LEfSe^29^) was carried out and a similar result was produced where *Prevotella 9* and *Bacteroides* were the top genera for enterotype 2 and 3 respectively in term of their differential abundance (Supplementary Data 3). On the other hand, LEfSe identified *Escherichia-Shigella* as the top genus in term of differential abundance for enterotype 1 followed by *Ruminococcaceae* UCG-002 and *Ruminococcaceae* UCG-010. This inconsistent result for enterotype 1 suggests that this enterotype may be less well defined by any single genus or could be further classified into smaller subgroups.

To further understand the potential influence of each enterotype’s main contributor in overall gut microbiota, a correlation analysis of co-occurring genera was carried out and the result is shown in Fig. 3d. For enterotype 1, the enriched *Ruminococcaceae* UCG-002 and UCG-005 genera appeared to strongly co-occur with other diverse genera such as *Megamonas*, *Butyricimonas*, and *Christensenellaceae* R-7 group. On the other hand, the abundances of the enriched *Prevotella 9* in enterotype 2 and *Bacteroides* in enterotype 3 did not show a positive correlation with the abundance of any other genera. Specifically, the presence of *Prevotella 9* in high relative abundance among enterotype 2 samples was found to negatively associated with the presence of genera including *Fusobacterium*, *Lachnospiraceae* NK4A136 group, and *Bacteroides*. Similarly, the high abundance of *Bacteroides* in enterotype 3 samples appeared to be negatively correlated with the abundance of *Eubacterium* ventriosum group, *Subdoligranulum*, *Akkermansia*, *Butyricimonas*, *Ruminiclostridium* 6, and *Ruminococcaceae* UCG-005 (Fig. 3d). The co-occurrence analysis results for enterotype 2 and 3 are consistent with the reduction in diversity found among these samples (Fig. 3b).

In term of MetS patients’ clinical properties, parameters including smoke frequency, alcohol consumption, and F/B ratio (both qPCR and metagenomics) were significantly different between enterotypes (Supplementary Table S3). Specifically, MetS patients classified as enterotype 2 appeared to have significantly higher smoke frequency at 0.61 ± 0.69 days per week (p = 0.041) when compared to other enterotypes (0.37 ± 0.54 and 0.24 ± 0.43 days per week in enterotype 1 and 3, respectively). Similarly, alcohol consumption was also found to be significantly higher among enterotype 2 patients at 0.86 ± 0.87 days per week (p = 0.001) relative to 0.34 ± 0.71 days per week in enterotype 1 and 0.27 ± 0.65 days per week in enterotype 3. On the other hand, F/B ratio was found to be significantly increased among enterotype 1 samples (0.89 ± 0.46, p = 0.007) compared to the other enterotypes (0.59 ± 0.16 and 0.67 ± 0.23 in enterotype 1 and 3 respectively). While variations could also be observed in other clinical parameters, their differences between enterotypes were not statistically significant (Supplementary Table S3). These include, for instance, age (enterotype 1: 64.45 ± 9.10, enterotype 2: 63.36 ± 7.58, and enterotype 3: 65.05 ± 8.62), BMI (enterotype 1: 27.35 ± 6.08, enterotype 2: 28.49 ± 5.65, and enterotype 3: 26.74 ± 5.56), HDL cholesterol (enterotype 1: 52.42 ± 19.54, enterotype 2: 48.24 ± 16.73, and enterotype 3: 48.23 ± 13.55), LDL cholesterol (enterotype 1: 100.84 ± 39.38, enterotype 2: 93.97 ± 39.24, and enterotype 3: 88.39 ± 28.95), glucose (enterotype 1: 130.86 ± 48.91, enterotype 2: 128.82 ± 44.82, and enterotype 3: 130.65 ± 41.62) and insulin (enterotype 1: 6.31 ± 5.49, enterotype 2: 8.64 ± 7.37, and enterotype 3: 9.06 ± 12.40).

### Correlation analysis between bacterial abundance and metabolic parameters

**Figure 4:**
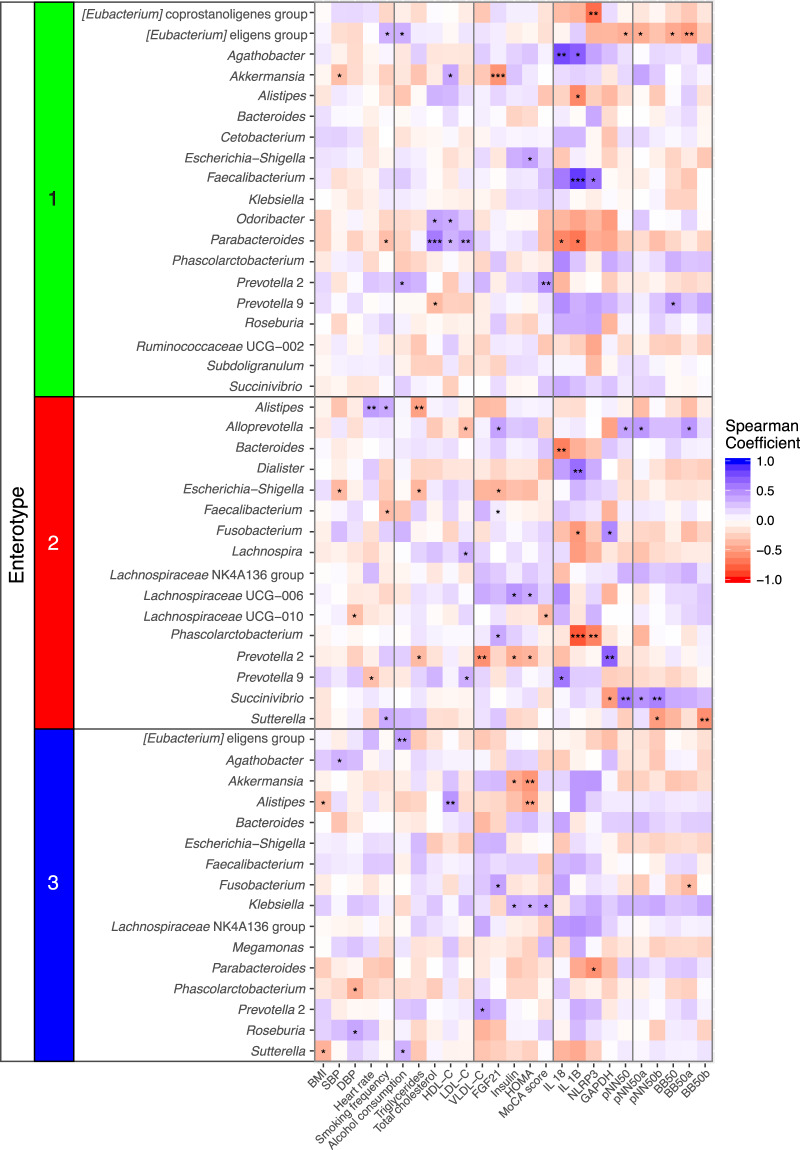
A heatmap showing the correlations between species abundance (rows) and clinical parameters (columns) for each enterotypes of metabolic syndrome patients. Positive correlations are shown using a gradient of blue colors. Negative correlations are shown using a gradient of red colors. Correlations with p-values that are statistically significant are noted by asterisk(s). See Methods section for meaning of all abbreviations.

Correlation analyses between each genus abundance and patient’s clinical parameters were performed separately for each group of MetS patients (enterotype 1, 2, and 3) to identify associations between host clinical parameters and bacterial abundances. The results are summarized in Fig. 4 and the detailed results of Fig. 4 are shown in Supplementary Data 4. Notably, the same correlation analyses were also carried out using all gut microbiota data (111 samples) and no strong association (r ≥ 0.4 or ≤ −0.4) between the abundance of any genus and clinical parameters were identified (Supplementary Data 5).

For enterotype 1 MetS patients, several significant associations were detected. Among all positive correlations, levels of interleukin 1 beta (IL 1B) and interleukin 18 (IL 18) showed the strongest associations with the abundance of *Faecalibacterium* (r = 0.839, p < 0.001) and *Agathobacter* (r = 0.781, p = 0.003) respectively. On the other hand, the abundance of *Parabacteroides* appeared to be associated to lipid profiles as the results showed significant positive correlations with parameters including total cholesterol (r = 0.570, p < 0.001), HDL-C (r = 0.331, p = 0.048), and low-density lipoprotein cholesterol (LDL-C) (r = 0.428, p = 0.008). Since these lipid related parameters are potentially collinear, we also analysed their correlations and found that total cholesterol and LDL-C were in fact highly correlated among enterotype 1 MetS patients (r = 0.878, p < 0.001) as shown in Supplementary Data 6. Among bacterial abundances that exhibit significant negative correlations with clinical parameters, *[Eubacterium] eligens group* appeared to be related to heart function as it is negatively correlated with several cardiac rhythm properties including percentage of intervals different from the preceding interval by at least 50 ms (pNN50) (r = −0.418, p = 0.030), percentage of intervals 50 ms longer than the preceding interval (pNN50a) (r = −0.426, p = 0.034), number of intervals different from the preceding interval by at least 50 ms (BB50) (r = −0.441, p = 0.015), and number of intervals at Least 50 ms Longer Than the Preceding Interval (BB50a) (r = −0.515, p = 0.004). However, we also note that correlation analyses of these cardiac rhythm properties showed that they are highly collinear with r values between 0.836 and 0.965 as shown in Supplementary Data 6.

Different correlation patterns between genus abundances and clinical parameters were found for enterotype 2 MetS patients as shown in Fig. 4. Of all the positive correlations, levels of Glyceraldehyde 3-phosphate dehydrogenase (GAPDH) and IL 1B exhibit the strongest association with the abundance of *Prevotella 2* (r = 0.690, p = 0.003) and *Dialister* (r = 0.632, p = 0.009) respectively. For this enterotype, the abundance of genus *Alloprevotella* showed the highest number of significant positive correlations with clinical parameters including Fibroblast growth factor 21 (FGF21) (r = 0.398, p = 0.029), pNN50 (r = 0.402, p = 0.042), pNN50a (r = 0.444, p = 0.039), and BB50a (r = 0.450, p = 0.027). While the cardiac rhythm values can be collinear against one another as observed in enterotype 1, they did not exhibit high collinearity with the Fibroblast growth factor 21 value (r values between 0.246 and 0.619). On the other hand, our analyses also reveal that some genera exhibit strong negative correlations with clinical parameters for this group of patients. Specifically, *Phascolarctobacterium* abundance was found to have negative correlations with IL 1B (r = −0.818, p = 0.001) and NOD-like receptor family, pyrin domain-containing protein 3 inflammasome (NLRP3) levels (r = −0.633, p = 0.009) where IL 1B and NLRP3 levels exhibit positive correlation of r = 0.715 (p = 0.002). Similarly, *Bacteroides* abundance showed a significant negative correlation with the IL 18 level (r = −0.645, p = 0.007).

For enterotype 3 MetS patients, a fewer number of significant correlations between genus abundances and clinical parameters were found when compared to other enterotypes (Fig. 4). Among these, genera such as *[Eubacterium] eligens group* and *Alistipes* exhibited strong positive correlations with alcohol consumption (r = 0.425, p = 0.009) and HDL-C (r = 0.459, p = 0.006) level respectively. Notably, genus *Klebsiella* appeared to have significant positive correlations with several clinical parameters including insulin level (r = 0.370, p = 0.037), Homeostatic Model Assessment (HOMA) score (r = 0.350, p = 0.049), and Montreal Cognitive Assessment (MoCA) score (r = 0.415, p = 0.016) where insulin value and HOMA score appeared to be collinear with one another (r = 0.934, p < 0.001). On the other hand, HOMA also appeared to be significantly associated with *Akkermansia* (r = −0.537, p = 0.002), and *Alistipes* (r = −0.479, p = 0.006) in a negative manner.

## Discussion

The major findings of this study are: (1) gut microbiota profiles of 111 treated MetS patients are highly diverse across individuals (beta diversity); (2) although gut microbiota profiles of these MetS patients are diverse, they can be classified into three groups or enterotypes based on the similarity of their genera compositions where enterotype 1 is identifiable by *Ruminococaceae* UCG-002 and UCG-005 abundances, enterotype 2 is identifiable by *Prevotella* 9 abundance, and enterotypes 3 is identifiable by *Bacteroides* abundance. (3) In relation to clinical parameters, enterotype 2 appeared to be significantly enriched in MetS patients with high levels of smoking frequency and alcohol consumption. (4) Many significantly strong associations between clinical parameters and genus abundances were found to be enterotype-specific. For instance, the IL 1B level was positively correlated with the abundance of *Faecalibacterium* in enterotype 1 group whereas the same parameter was positively correlated with *Dialister* in enterotype 2 group.

Metabolic syndrome (MetS) has become a global public health issue. Previous studies have shown that gut microbiota plays an important role in the pathogenesis of this disease^30–33^. Better understanding of gut microbiota variability among clinically-treated MetS patients can potentially be useful in assessing and classifying patients as well as informing more suitable treatment options. Our result revealed that gut microbiota of treated MetS patients can be classified into three distinct enterotypes (Fig. 2) similar to the previously published result of human enterotypes by Arumugam *et al*.^24^. The differential abundance analysis results show that enterotype 1 group is enriched with *Ruminococcaceae*, a genus that has been linked to the reduction of BMI^34^. On the other hand, enterotype 2 group was enriched with genus *Prevotella 9*. Comparisons of these *Prevotella 9* sequences with the NCBI database using BLAST shows that *Prevotella copri* DSM 18205 was repeatedly the best hit (387 out of 390 sequences) with 96.59% average pairwise sequence similarity (Supplementary Data 7). *Prevotella* has been shown to be a large genus that is made up of very genetically diverse species^35,36^. In addition, presence of *Prevotella copri* has been reported to increase the risk of diarrhea and irritable bowel syndrome^37,38^. Lastly, genera including *Bacteroides*, *Hungatella*, and *Akkermensia* were enriched in gut microbiota of enterotype 3 MetS patients. Genus *Akkermensia* contains probiotic bacterial species such as *Akkermansia muciniphila*^39^, which has previously been linked to mucus layer-protection and is known to possess potential anti-inflammatory properties, both of which can improve gut function^40^. Further comparisons of *Akkermensia* sequences from this enterotype against NCBI database showed that *Akkermansia muciniphila* was repeatedly the best hit (64 out of 66 sequences) with 95.13% average of pairwise sequence similarity (Supplementary Data 8). The enrichment of this bacterium may be beneficial to enterotype 3 MetS patients. *Bifidobacterium* spp., which is a known beneficial bacterium^41^, was not significantly enriched in any of the three groups of MetS patients (Supplementary Data 2 and Data 3).

Most parameters that are directly related to MetS (such as BMI, LDL-C, triglyceride, glucose, and insulin) are not significantly different between the three enterotypes (Supplementary Table S3). This could be due to the fact that MetS patients in this study were clinically treated and their metabolic parameters were well controlled with their medications. However, our results show that enterotype 2 MetS patients have significantly higher alcohol consumption and smoking frequency when compared to patients in another enterotypes. This result may suggest potential relationship between these behaviors and the enrichment of genus *Prevotella 9* in these patients.

To better understand the possible effects of gut microbiota on patient physiology, correlation analyses between each genus abundance and clinical parameters were carried out. When the analysis was performed using the microbiota profile of all 111 MetS patients at both phylum and genus levels, we did not find any significantly strong correlation (r ≥ 0.4 or ≤ −0.4) between taxon and clinical parameters. However, when the analyses were done separately for each group of patients, many interesting correlations were identified as summarized in Fig. 4 and Supplementary Data 4. Notably, the same genus abundance may show significant positive or negative correlations with different metabolic parameters in different enterotypes of MetS patients. For instance, our result shows that the genus *Faecalibacterium* exhibited a strong significant positive correlation with the IL 1B level in enterotype 1 group while it exhibits a significant negative correlation with smoking frequency in enterotype 2 group. This example shows that the correlations between each genus abundance and clinical parameters may potentially be enterotype/group specific. On the other hand, it is also possible that the same genus presented in different groups may represent different bacterial species or strains. More detailed analyses using an approach such as whole-genome shotgun metagenomic sequencing to more accurately determine bacterial species/strains as well as functional genes that are present in the gut microbiota of MetS patients will be required to better understand these microbes-host correlations.

In summary, we have shown that gut microbiota profiles may be useful for classifying MetS patients. We have also described examples where several correlations between microbial abundance and patients’ clinical parameters are group/enterotype specific. Despite these results, we note that our study does contain some limitations. Since, our results are based on 16S rRNA metagenomics data of a cohort from the same region (Northern part of Thailand), usage of the findings reported here must be proceeded with cautions. More studies based on a larger and ethnically different population using whole-genome shotgun metagenomic sequencing and metabolomics approaches should be carried out to further explore variations at functional levels (e.g. genes, and metabolites) within the gut microbial community to better understand their relationship with host parameters. While several enterotype-specific associations between individual genera and clinical parameters that we discovered may be relevant for personalised treatment, the mechanisms driving these associations are still unclear and our current data cannot delineate the exact cause-effect of these relationships. In addition, we note that while enterotype concept has been previously demonstrated to be useful in analysing the complex human gut microbiota, Costea and colleagues, for instance, has shown the controversy of this concept and standardization of this approach is still an active area of research^25^.

## Methods

### Subjects selection

The study protocol was approved by the institutional Ethics Committee of the Faculty of Medicine, Chiang Mai University, Chiang Mai, Thailand. Written informed consents were obtained from all patients (n = 111) prior to the study. All methods were performed in accordance with the relevant guidelines and regulations. This study is a sub-study of metabolic syndrome (MetS) patients in The Cohort Of patients at a high Risk for Cardiovascular Events (CORE) Thailand registry, which is an ongoing prospective cohort of Thai patients with a high atherosclerotic risk. Patients were recruited from outpatient clinic at Maharaj Nakorn Chiang Mai Hospital during the period between April 2011 and March 2014 using these criteria: 1) 45 years or older and 2) diagnosed with coronary artery disease (CAD), cerebrovascular disease (CVD), peripheral arterial disease (PAD), or at least 3 atherosclerosis risk factors (multiple risk factors, MRF). Some patients were excluded from the cohort for the following reasons: had an acute atherosclerotic event within 3 months, had a large aortic aneurysm indicated for surgery, participating in a blinded clinical trial, had a limited life expectancy from a non-cardiovascular condition such as cancer or a documented human immunodeficiency virus (HIV) infection, or might have difficulty returning for a follow-up visit. Patients were invited to participate in this study when they met three or more of the following five criteria for metabolic syndrome: elevated waist circumference (≥90 cm in men and ≥80 cm in women), blood pressure ≥130/85 mmHg or treated, triglycerides ≥150 mg/dl or treated, high density lipoprotein cholesterol (HDL-C) (<40 mg/dl in men and <50 mg/dl in women), and fasting glucose ≥100 mg/dl or treated^26^.

### Data and sample collection

MetS patients were first medically treated to control their metabolic symptoms before clinical data and sample collections were performed. All data and sample collections were carried out on the same day. These include history taking, physical examination, blood sampling and stool collection. The homeostasis model assessment (HOMA) index was used to assess the severity of peripheral insulin resistance as previously described^42^. Each patient’s stool sample was collected in a sterile plastic cup and kept in a refrigerator before sending to the Maharaj Nakorn Chiang Mai hospital within 24 hours.

### Metabolic parameter determination

Evaluation and calculation of the fasting plasma glucose, high-density lipoprotein cholesterol (HDL-C), low-density lipoprotein cholesterol (LDL-C), triglyceride, insulin and fibroblast growth factor 21 (FGF21) levels were carried out for all fasting blood samples that were obtained from participants. Colorimetric assay (ERBA diagnostic, Mannheim, Germany) was used to determine the fasting plasma glucose and triglyceride levels. A sandwich enzyme-linked immunosorbent assay (ELISA) kit (Millipore, MI, USA) was used to evaluate the fasting plasma insulin levels.

### DNA extraction

A commercial genomic DNA isolation kit (QIAGEN, Germany) was used to extract bacterial genomic DNA from patient fecal samples. Briefly, the fecal sample (0.25 g) was homogenized in QIAGEN ASL lysis buffer using a Minibeadbeater (BioSpec Products, Bartlesville, US). Then, following the manufacturer’s instructions, the bacterial genomic DNA was extracted from the feces. The fractions of bacterial microbiota population representing the Firmicutes/Bacteroidetes (F/B) ratio were quantified using quantitative polymerase chain reaction (qPCR) as described previously^43,44^.

### PCR amplification and sequencing of the 16S rRNA Gene

Amplification of the V3–V4 hypervariable region of the microbial 16S rRNA gene was performed using the universal primers 341 F, 5′ACACTGACGACATGGTTCTACACCTACGGGNGGCWGCAG-3′ and 805 R, 5′-TACGGTAGCAGAGACTTGGTCTGACTACHVGGGTATCTAATCC-3′. Amplification consisted of the following steps: an initial denaturing at 94 °C for 3 minutes followed by 35 cycles of 94 °C for 45 seconds, 50 °C for 1 minute, 72 °C for 90 seconds, and an elongation step of 72 °C for 10 minutes^45^. After PCR amplification, paired-end sequencing was performed using the Illumina MiSeq platform at Génome Québec, Canada.

### Gut microbiota analysis

The raw paired-end sequences were analysed by using the Quantitative Insights Into Microbial Ecology 2 (QIIME2; version 2019.7.0) package^46,47^. The DADA2 pipeline within QIIME2 was used to filter low-quality reads, denoise sequences, join paired-end reads, and remove chimeric sequences^48^. The taxonomy was assigned using the scikit-learn naive Bayes machine-learning classifier^49^ where the classifier was created by using sequences from the SILVA database version 132^50^. Rarefying normalization^51^ was applied to normalize all samples using the 15,000 reads per sample criterion. To group samples based on similar microbiota profiles at genus level, a clustering method previously described by Arumugam *et al*. was applied^24^. Briefly, the partitioning around medoid (PAM) clustering algorithm with Jensen–Shannon distance was employed to cluster samples based on relative genus abundances where the optimal number of clusters was determined by using Calinski–Harabasz (CH) index. The clustering result was then used to assign samples into groups that share similar relative genus abundances (enterotypes). Alpha diversity of each enterotype was determined using Shannon index. Finally, the enterotypes of all MetS samples were visualized using principal component analysis.

### Statistical analysis

Continuous variables are expressed as mean ± standard deviation. The analysis of non-normally distributed variables was done using the Mann-Whitney U and Kruskal-Wallis Tests^52,53^. Frequencies and percentages are used for all of the categorical variables. Pearson χ² test was used to compare between groups. The differential abundance test between groups was done using analysis of composition of microbiomes (ANCOM) and linear discriminant analysis effect size (LEfSe)^28,29^. The metadata of samples used in this study consisted of the non-normally distributed variables such as age, height, weight, body mass index (BMI), waist circumference, smoking frequency, alcohol consumption frequency, high-density lipoprotein cholesterol (HDL-C), low-density lipoprotein cholesterol (LDL-C), very-low-density lipoprotein cholesterol (VLDL-C), heart rate, systolic blood pressure (SBP), diastolic blood pressure (DBP), triglyceride, total cholesterol, glucose, insulin, homeostatic model assessment (HOMA) score, Montreal cognitive assessment (MoCA) score, interleukin 18 (IL 18), interleukin 1 beta (IL 1B), NOD-like receptor family, pyrin domain-containing protein 3 inflammasome (NLRP3), and glyceraldehyde 3-phosphate dehydrogenase (GAPDH). Cardiovascular disease and co-morbidities as well as cardiac rhythms were also recorded. The correlations between the microbial abundances and clinical parameters were analysed using Spearman’s rank correlation coefficient^54^. Note that species with abundance of less than 1 percent were excluded from the correlation analysis. Spearman’s rank correlation was also used to compare clinical parameters to identify collinearity between parameter values. P values of less than 0.05 based on 2-tailed probability tests were regarded as statistically significant. All statistical calculations were performed using the SPSS version 17 (SPSS Inc., IL, USA).

## Supplementary information

Supplemental information.

Dataset 1.

Dataset 3.

Dataset 2.

Dataset 4.

Dataset 5.

Dataset 6.

Dataset 7.

Dataset 8.

## Data Availability

The datasets used in this study are available from the corresponding authors on reasonable request.
