# Supplementary material for: Gut Microbiota Profiles of Treated Metabolic Syndrome Patients and their Relationship with Metabolic Health
Source: Sci Rep. 2020 Jun 22;10:10085. doi: 10.1038/s41598-020-67078-3 (PMC7308281; doi:10.1038/s41598-020-67078-3)
Supplement: Supplementary file 1 — Supplemental information. [file 41598_2020_67078_MOESM1_ESM.docx]

**Gut Microbiota Profiles of Treated Metabolic Syndrome Patients and their Relationship with Metabolic Health**

Montree Wutthi-in^1,2^, Supapon Cheevadhanarak^2,3^, Sakawdaurn Yasom^4,5,6^, Sasiwan Kerdphoo^4,5^, Parameth Thiennimitr^4,5,6^, Arintaya Phrommintikul^4,5,7^, Nipon Chattipakorn^4,5^, Weerayuth Kittichotirat^1,2*^, Siriporn Chattipakorn^4,5,8*^

^1^ Bioinformatics and Systems Biology Program, School of Bioresources and Technology and School of Information Technology, King Mongkut’s University of Technology Thonburi, Bangkok 10150, Thailand; ^2^ Systems Biology and Bioinformatics Research Group, Pilot Plant Development and Training Institute, King Mongkut's University of Technology Thonburi, Bangkok 10150, Thailand; ^3^ School of Bioresources and Technology, King Mongkut's University of Technology Thonburi, Bangkok 10150, Thailand; ^4^ Cardiac Electrophysiology Research and Training Center, Faculty of Medicine, Chiang Mai University, Chiang Mai 50200, Thailand; ^5^ Center of Excellence in Cardiac Electrophysiology Research, Faculty of Medicine, Chiang Mai University, Chiang Mai 50200, Thailand;  ^6^ Department of Microbiology, Faculty of Medicine, Chiang Mai University, Chiang Mai, 50200, Thailand; ^7^ Department of Internal Medicine, Faculty of Medicine, Chiang Mai University, Chiang Mai, 50200, Thailand;^8^ Department of Oral Biology and Diagnostic Sciences, Faculty of Dentistry, Chiang Mai University, Chiang Mai, 50200, Thailand

**Corresponding authors**

*Weerayuth Kittichotirat, PhD.

Systems Biology and Bioinformatics Research Group, Pilot Plant Development and Training Institute, King Mongkut's University of Technology Thonburi, Bangkok 10140, Thailand

Tel: +66-2-470-7407, Fax: +66-2-452-3455

Email: weerayuth.kit@kmutt.ac.th; weerayuth@gmail.com

*Siriporn C. Chattipakorn, DDS, PhD

Neurophysiogy Unit, Cardiac Electrophysiology Research and Training Center, Faculty of Medicine, Chiang Mai University, and Department of Oral Biology and Diagnostic Science, Faculty of Dentistry, Chiang Mai University, Chiang Mai, 50200, Thailand.

Tel: 011-66-53-944-451, Fax: 011-66-53-222-844

Email: scchattipakorn@gmail.com; siriporn.c@cmu.ac.th

**
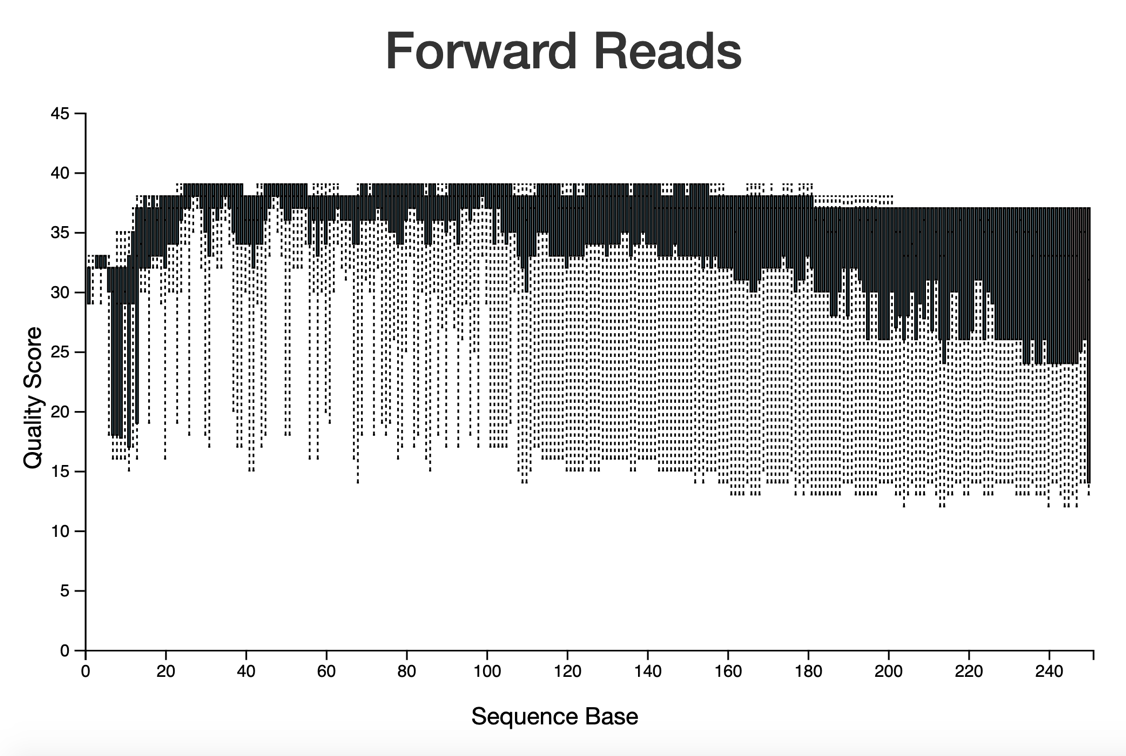
**


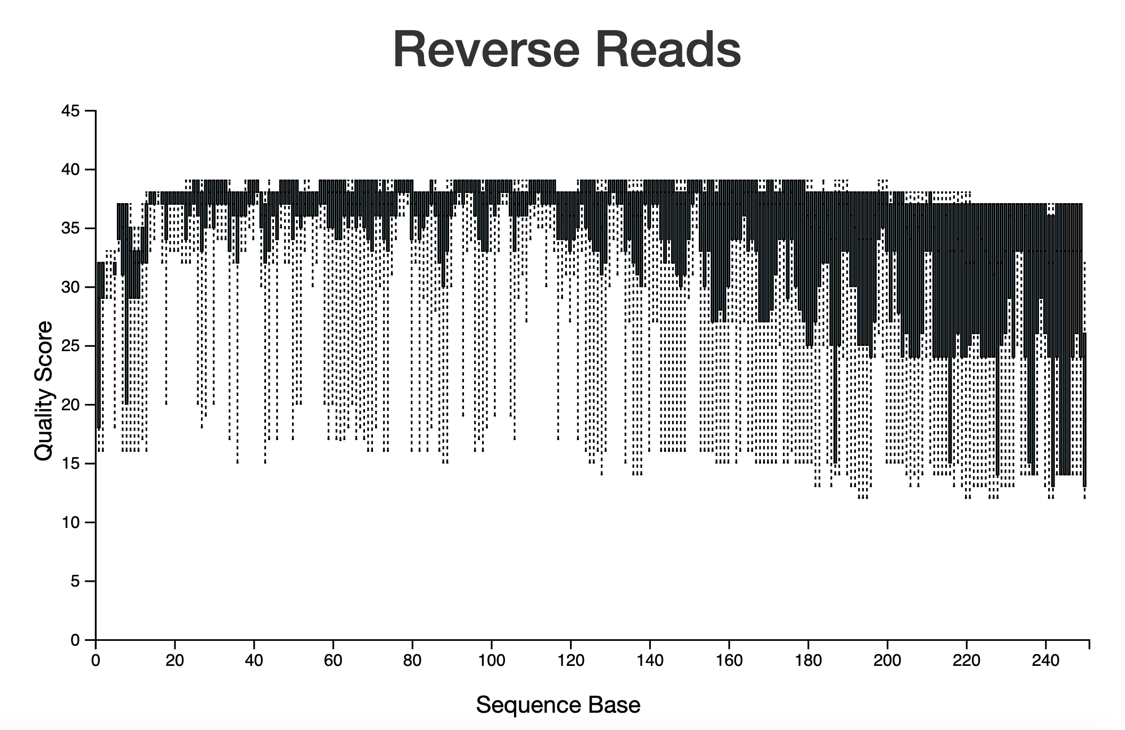


**Supplementary Figure S1.** Average sequence base quality of forward (Top) and reverse (Bottom) reads in each position. This figure was created by using 10,000 random sequences from every sample.

**Supplementary Figure S2.** The estimation of optimal number of clusters (enterotypes) using Calinski-Harbasz (CH) index. This figure shows that 3 clusters (enterotypes) is optimal cluster for our dataset.

**Supplementary Table S1.** Number of sequencing reads for each sample.

| **Sample ID** | **Number of reads** |
| --- | --- |
| NC-001 | 67,242 |
| NC-002 | 55,532 |
| NC-003 | 56,778 |
| NC-005 | 59,669 |
| NC-006 | 64,087 |
| NC-007 | 65,533 |
| NC-008 | 71,919 |
| NC-009 | 58,931 |
| NC-010 | 69,367 |
| NC-011 | 59,535 |
| NC-012 | 60,672 |
| NC-013 | 58,915 |
| NC-020 | 71,620 |
| NC-021 | 63,917 |
| NC-022 | 65,395 |
| NC-023 | 48,628 |
| NC-024 | 69,113 |
| NC-025 | 59,864 |
| NC-026 | 63,647 |
| NC-027 | 70,174 |
| NC-029 | 69,226 |
| NC-031 | 71,277 |
| NC-033 | 68,382 |
| NC-034 | 53,728 |
| NC-035 | 67,735 |
| NC-036 | 60,227 |
| NC-037 | 62,549 |
| NC-038 | 68,051 |
| NC-039 | 67,304 |
| NC-040 | 62,970 |
| NC-041 | 60,358 |
| NC-042 | 62,347 |
| NC-043 | 79,521 |
| NC-044 | 60,025 |
| NC-045 | 71,461 |
| NC-046 | 65,352 |
| NC-047 | 64,769 |
| NC-048 | 60,466 |
| NC-049 | 64,444 |
| NC-050 | 58,816 |
| NC-051 | 69,031 |
| NC-052 | 56,756 |
| NC-053 | 61,997 |
| NC-054 | 63,723 |
| NC-055 | 66,697 |
| NC-056 | 55,005 |
| NC-057 | 60,448 |
| NC-058 | 67,009 |
| NC-059 | 66,955 |
| NC-060 | 71,212 |
| NC-061 | 67,433 |
| NC-062 | 66,083 |
| NC-063 | 60,968 |
| NC-064 | 68,645 |
| NC-065 | 62,822 |
| NC-066 | 71,667 |
| NC-067 | 67,652 |
| NC-068 | 67,033 |
| NC-069 | 62,596 |
| NC-070 | 77,369 |
| NC-071 | 81,177 |
| NC-072 | 59,285 |
| NC-073 | 80,622 |
| NC-074 | 74,747 |
| NC-075 | 79,953 |
| NC-076 | 75,119 |
| NC-077 | 36,400 |
| NC-078 | 64,364 |
| NC-079 | 59,986 |
| NC-080 | 73,416 |
| NC-081 | 60,327 |
| NC-082 | 62,363 |
| NC-083 | 76,002 |
| NC-084 | 68,800 |
| NC-085 | 60,397 |
| NC-086 | 45,388 |
| NC-087 | 61,498 |
| NC-089 | 63,482 |
| NC-090 | 70,590 |
| NC-092 | 43,483 |
| NC-093 | 74,519 |
| NC-094 | 83,728 |
| NC-095 | 61,525 |
| NC-096 | 65,851 |
| NC-097 | 82,472 |
| NC-098 | 76,527 |
| NC-099 | 82,248 |
| NC-100 | 82,571 |
| NC-101 | 73,327 |
| NC-102 | 72,690 |
| NC-103 | 74,303 |
| NC-104 | 74,058 |
| NC-105 | 74,436 |
| NC-106 | 68,593 |
| NC-107 | 68,616 |
| NC-108 | 68,201 |
| NC-109 | 75,000 |
| NC-110 | 68,811 |
| NC-111 | 64,370 |
| NC-112 | 68,868 |
| NC-113 | 73,961 |
| NC-114 | 64,125 |
| NC-115 | 66,160 |
| NC-116 | 53,760 |
| NC-118 | 50,832 |
| NC-119 | 82,570 |
| NC-120 | 75,011 |
| NC-121 | 69,963 |
| NC-122 | 77,798 |
| NC-124 | 63,457 |
| NC-125 | 80,053 |
| NC-126 | 65,724 |

**Supplementary Table S2.** Sample ID and enterotype assignment

| **Sample ID** | **Enterotypes ID** |
| --- | --- |
| NC-001 | 1 |
| NC-002 | 2 |
| NC-003 | 2 |
| NC-005 | 2 |
| NC-006 | 2 |
| NC-007 | 1 |
| NC-008 | 3 |
| NC-009 | 2 |
| NC-010 | 2 |
| NC-011 | 2 |
| NC-012 | 3 |
| NC-013 | 1 |
| NC-020 | 1 |
| NC-021 | 2 |
| NC-022 | 3 |
| NC-023 | 3 |
| NC-024 | 2 |
| NC-025 | 3 |
| NC-026 | 3 |
| NC-027 | 3 |
| NC-029 | 1 |
| NC-031 | 3 |
| NC-033 | 2 |
| NC-034 | 2 |
| NC-035 | 1 |
| NC-036 | 1 |
| NC-037 | 1 |
| NC-038 | 3 |
| NC-039 | 1 |
| NC-040 | 1 |
| NC-041 | 2 |
| NC-042 | 3 |
| NC-043 | 1 |
| NC-044 | 3 |
| NC-045 | 3 |
| NC-046 | 1 |
| NC-047 | 3 |
| NC-048 | 1 |
| NC-049 | 3 |
| NC-050 | 1 |
| NC-051 | 1 |
| NC-052 | 3 |
| NC-053 | 2 |
| NC-054 | 2 |
| NC-055 | 1 |
| NC-056 | 3 |
| NC-057 | 1 |
| NC-058 | 1 |
| NC-059 | 1 |
| NC-060 | 1 |
| NC-061 | 3 |
| NC-062 | 1 |
| NC-063 | 3 |
| NC-064 | 1 |
| NC-065 | 3 |
| NC-066 | 2 |
| NC-067 | 1 |
| NC-068 | 2 |
| NC-069 | 3 |
| NC-070 | 2 |
| NC-071 | 3 |
| NC-072 | 2 |
| NC-073 | 2 |
| NC-074 | 1 |
| NC-075 | 1 |
| NC-076 | 2 |
| NC-077 | 3 |
| NC-078 | 3 |
| NC-079 | 3 |
| NC-080 | 3 |
| NC-081 | 2 |
| NC-082 | 3 |
| NC-083 | 2 |
| NC-084 | 1 |
| NC-085 | 2 |
| NC-086 | 3 |
| NC-087 | 3 |
| NC-089 | 3 |
| NC-090 | 1 |
| NC-092 | 3 |
| NC-093 | 1 |
| NC-094 | 1 |
| NC-095 | 2 |
| NC-096 | 1 |
| NC-097 | 2 |
| NC-098 | 2 |
| NC-099 | 1 |
| NC-100 | 1 |
| NC-101 | 1 |
| NC-102 | 3 |
| NC-103 | 2 |
| NC-104 | 2 |
| NC-105 | 2 |
| NC-106 | 2 |
| NC-107 | 3 |
| NC-108 | 1 |
| NC-109 | 1 |
| NC-110 | 1 |
| NC-112 | 2 |
| NC-113 | 2 |
| NC-114 | 1 |
| NC-115 | 1 |
| NC-116 | 3 |
| NC-118 | 3 |
| NC-119 | 3 |
| NC-120 | 3 |
| NC-121 | 2 |
| NC-122 | 3 |
| NC-124 | 2 |
| NC-125 | 2 |
| NC-126 | 2 |

**Supplementary Table S3.** Differences in patient’s clinical parameters between enterotypes using Kruskal-Wallis test. Parameters that are statistically significant are denoted by asterisk(s).

| **Clinical parameters** | **Enterotype 1** | **Enterotype 2** | **Enterotype 3** | **p-value** |
| --- | --- | --- | --- | --- |
| Age | 64.45 ± 9.10 | 63.36 ± 7.58 | 65.05 ± 8.62 | 0.627 |
| Height | 155.87 ± 6.31 | 158.94 ± 7.19 | 157.73 ± 8.31 | 0.229 |
| Weight | 66.66 ± 16.27 | 71.82 ± 13.57 | 66.74 ± 15.55 | 0.113 |
| BMI | 27.35 ± 6.08 | 28.49 ± 5.65 | 26.74 ± 5.56 | 0.235 |
| Waist circumference | 93.95 ± 15.00 | 98.26 ± 12.82 | 92.71 ± 11.82 | 0.222 |
| Systolic blood pressure | 141.42 ± 17.41 | 136.17 ± 17.23 | 136.16 ± 17.78 | 0.295 |
| Diastolic blood pressure | 74.82 ± 7.94 | 73.22 ± 10.30 | 70.03 ± 10.02 | 0.08 |
| Pulse | 77.68 ± 14.12 | 72.36 ± 11.66 | 72.30 ± 9.72 | 0.085 |
| Smoke frequency | 0.37 ± 0.54 | 0.61 ± 0.69 | 0.24 ± 0.43 | 0.041* |
| Alcohol consumption | 0.34 ± 0.71 | 0.86 ± 0.87 | 0.27 ± 0.65 | 0.001** |
| Triglyceride | 133.91 ± 82.60 | 127.42 ± 52.93 | 138.66 ± 83.16 | 0.829 |
| Total cholesterol | 161.28 ± 39.42 | 156.91 ± 43.74 | 150.67 ± 40.07 | 0.518 |
| High-density lipoprotein cholesterol | 52.42 ± 19.54 | 48.24 ± 16.73 | 48.23 ± 13.55 | 0.495 |
| Low-Density lipoprotein cholesterol | 100.84 ± 39.38 | 93.97 ± 39.24 | 88.39 ± 28.95 | 0.407 |
| Very-low-density lipoprotein cholesterol | 27.11 ± 16.59 | 27.10 ± 11.23 | 30.13 ± 18.56 | 0.691 |
| Glucose | 130.86 ± 48.91 | 128.82 ± 44.82 | 130.65 ± 41.62 | 0.942 |
| Hemoglobin A1C | 7.48 ± 1.72 | 6.91 ± 1.33 | 7.08 ± 1.27 | 0.469 |
| Fibroblast growth factor 21 | 407.24 ± 373.96 | 351.70 ± 253.35 | 407.52 ± 769.75 | 0.758 |
| Insulin | 6.31 ± 5.49 | 8.64 ± 7.37 | 9.06 ± 12.40 | 0.226 |
| Homeostatic Model Assessment score | 2.46 ± 2.95 | 2.44 ± 1.61 | 2.89 ± 4.18 | 0.387 |
| Montreal Cognitive Assessment score | 19.12 ± 4.70 | 19.17 ± 3.58 | 20.39 ± 5.19 | 0.324 |
| Interleukin-18 | 662.06 ± 487.00 | 539.07 ± 280.20 | 538.28 ± 274.79 | 0.934 |
| Interleukin 1 beta | 569.46 ± 303.70 | 434.58 ± 292.76 | 480.35 ± 379.86 | 0.401 |
| NOD-, LRR- and pyrin domain-containing protein 3 | 2.05 ± 0.58 | 1.73 ± 0.68 | 1.74 ± 0.63 | 0.195 |
| Glyceraldehyde 3-phosphate dehydrogenase | 1.09 ± 0.26 | 1.15 ± 0.10 | 1.09 ± 0.30 | 0.451 |
| pNN50 | 9.81 ± 14.14 | 5.17 ± 8.20 | 9.16 ± 15.64 | 0.487 |
| pNN50a | 4.83 ± 6.74 | 2.89 ± 4.54 | 4.36 ± 7.33 | 0.828 |
| pNN50b | 5.50 ± 7.72 | 2.81 ± 4.04 | 5.67 ± 8.87 | 0.463 |
| BB50 | 48.17 ± 49.67 | 50.00 ± 54.07 | 67.03 ± 100.95 | 0.976 |
| BB50a | 22.79 ± 25.06 | 22.63 ± 22.85 | 32.37 ± 47.63 | 0.945 |
| BB50b | 27.93 ± 24.50 | 32.28 ± 36.85 | 42.81 ± 57.00 | 0.991 |
| F/B ratio (16S rRNA) | 0.89 ± 0.46 | 0.59 ± 0.16 | 0.67 ± 0.23 | 0.007** |
| F/B ratio (qPCR) | 0.63 ± 0.54 | 0.34 ± 0.30 | 0.49 ± 0.32 | 0.007** |
